# Reply to the Letter to the Editor: Separating LLM repeatability from expert concordance in radiomics quality assessment

**DOI:** 10.1186/s41747-026-00859-z

**Published:** 2026-11-13

**Authors:** Ludwig Federico Garello, Valentina Giannini, Marco Gatti, Arianna Defeudis, Debora Cafaro, Giulia Nicoletti, Noemi Cristina Culasso, Andrea Veltri, Renato Cuocolo, Maurizio Balbi

**Affiliations:** 1 Radiology Unit, San Luigi Gonzaga Hospital, Orbassano, Turin, Italy; 2 Department of Oncology, University of Turin, Orbassano, Turin, Italy; 3 Department of Surgical Sciences, University of Turin, Turin, Italy; 4 Azienda Ospedaliero-Universitaria Città della Salute e della Scienza di Torino, Turin, Italy; 5 Candiolo Cancer Institute, FPO-IRCCS, Candiolo, Turin, Italy; 6 Department of Medicine, Surgery and Dentistry, University of Salerno, Baronissi, Italy

Dear Editor,

We thank Yang, Meng, and Shen for their thoughtful comments on our study and for highlighting the distinction between agreement with human readers and within-model repeatability in large language model (LLM)-based radiomics quality assessment.

We agree that intra-model repeatability is an important dimension of LLM reliability, distinct from human–LLM agreement. In our study, each article was assessed once by ChatGPT 5.1 Thinking using standardized prompts. A separate temporary session with memory disabled was used for each prompt to minimize carry-over effects. As repeated assessments of the same article were not performed, run-to-run variability could not be retrospectively quantified. We aimed to explore the performance of ChatGPT 5.1 Thinking in applying METRICS and RQS and to assess its agreement with human evaluations in a single-pass setting, rather than to comprehensively assess LLM reliability [1]. Accordingly, the reported ICCs should be interpreted as measures of human–LLM agreement and not as evidence of intra-model repeatability. Repeated LLM assessments would enable evaluation of this additional aspect of reliability in future validation studies [2].

We would also like to clarify the structure of the human assessments. As described in our “Methods” section, two readers with complementary expertise generated the main reader assessment, each independently scoring items in their domain, with item-level scores subsequently combined into a single assessment. We used the same approach for secondary reader groups A and B, without consensus meetings. Thus, the ICC analyses compared ChatGPT with composite expert assessments rather than with individual readers completing all METRICS and RQS items. Because no individual reader independently scored all items, agreement between ChatGPT and individual readers for the overall METRICS and RQS scores cannot be derived from the current dataset [1].

We also appreciate the authors’ observation regarding the ICC specification. We selected an absolute-agreement, single-measure formulation because systematic differences between scores were treated as disagreement, and each comparison was based on a single score from each assessment configuration rather than the mean of multiple assessments [1]. Given that the human reader groups and the ChatGPT model and prompting strategy were predefined, the resulting ICCs should be interpreted as agreement between the specific assessment configurations evaluated, rather than as estimates generalizable to other human readers, LLMs, or prompting strategies, as outlined by Koo and Li [3]. For the same data, the corresponding two-way mixed-effects, absolute-agreement, single-measure formulation yields the same numerical ICC point estimate as the two-way random-effects formulation [4]. Consequently, this distinction affects interpretation and generalizability rather than the reported ICC estimates [3].

Finally, we agree that model version and inference conditions should be documented as completely as permitted by the platform used. ChatGPT 5.1 Thinking was accessed through the web application on 8 December 2025. Sampling parameters (*e.g*., temperature) were not user-configurable through this interface [1]. Future studies should, where technically feasible, preserve repeated run-level outputs and report relevant model and generation parameters to facilitate reproducibility and allow stochastic variability to be distinguished from systematic scoring differences.

These considerations provide important context for the interpretation of our findings. Our study explored agreement between ChatGPT 5.1 Thinking and composite expert assessments within the specific evaluation framework used but did not evaluate within-model repeatability. Accordingly, the item-level discrepancies observed in our study should be regarded as descriptive patterns of the single-pass assessments rather than as evidence of stable bias across repeated runs. This remains consistent with our cautious conclusion that ChatGPT 5.1 Thinking may assist radiomics quality assessment, while discrepancies with human evaluations warrant caution [1]. We thank Yang and colleagues for raising this important methodological point and for the opportunity to further clarify the scope of our findings.

## Data Availability

Data sharing does not apply to this article as no datasets were generated or analyzed during the current study.
